# Synthesis, Characterization, and Antimicrobial Activity of Magnesium-Doped Hydroxyapatite Suspensions

**DOI:** 10.3390/nano9091295

**Published:** 2019-09-11

**Authors:** Daniela Predoi, Simona Liliana Iconaru, Mihai Valentin Predoi, George E. Stan, Nicolas Buton

**Affiliations:** 1 National Institute of Materials Physics, 405A Atomistilor Street, P.O. Box MG7, 077125 Magurele, Romania; simonaiconaru@gmail.com (S.L.I.); george_stan@infim.ro (G.E.S.); 2 University Politehnica of Bucharest, BN 002, 313 Splaiul Independentei, Sector 6, 10023 Bucharest, Romania; predoi@gmail.com; 3 HORIBA Jobin Yvon S.A.S., 6–18, Rue du Canal, 91165 Longjumeau CEDEX, France; nicolas.buton@horiba.com

**Keywords:** magnesium, hydroxyapatite, suspensions, ultrasound measurements, antimicrobial activity

## Abstract

Obtaining nanoscale materials has allowed for the miniaturization of components, which has led to the possibility of achieving more efficient devices with faster functions and much lower costs. While hydroxyapatite [HAp, Ca_10_(PO_4_)_6_(OH)_2_] is considered the most widely used material for medical applications in orthopedics, dentistry, and general surgery, the magnesium (Mg) is viewed as a promising biodegradable and biocompatible implant material. Furthermore, Mg is regarded as a strong candidate for developing medical implants due to its biocompatibility and antimicrobial properties against gram-positive and gram-negative bacteria. For this study, magnesium-doped hydroxyapatite (Ca_10−x_Mg_x_ (PO_4_)_6_ (OH)_2_, x_Mg_ = 0.1), 10MgHAp, suspensions were successfully obtained by an adapted and simple chemical co-precipitation method. The information regarding the stability of the nanosized 10MgHAp particles suspension obtained by ζ-potential analysis were confirmed for the first time by a non-destructive ultrasound-based technique. Structural and morphological studies of synthesized 10MgHAp were conducted by X-ray diffraction (XRD), Fourier-transform infrared (FTIR) spectroscopy in attenuated total reflectance (ATR) mode and scanning electron microscopy (SEM). The XRD analysis of the 10MgHAp samples confirmed that a single crystalline phase associated to HAp with an average grain size about 93.3 nm was obtained. The FTIR-ATR spectra revealed that the 10MgHAp sample presented broader IR bands with less visible peaks when compared to a well-crystallized pure HAp. The SEM results evidenced uniform MgHAp nanoparticles with spherical shape. The antimicrobial activity of the 10MgHAp suspension against gram-positive strains (*Staphylococcus aureus* ATCC 25923, *Enterococcus faecalis* ATCC 29212), gram-negative strains (*Escherichia coli* ATCC 25922, *Pseudomonas aeruginosa* ATCC 27853), as well as a fungal strain (*Candida albicans* ATCC 90029) were evaluated.

## 1. Introduction

Nowadays, bone substitutions are being increasingly used in surgery and are reported in second place in the human tissue replacement procedures after blood transfusion. More than two million bone grafting procedures are performed annually at a global scale. Usually, bone tissue replacements are synthesized from bioactive materials that possess similarities with the natural human bone [1,2]. The materials generally used as bone substitutes are the biocompatible ceramics that have a mechanical strength similar to that of human bone. A category of materials that have the capacity to stimulate bone reconstruction are the calcium phosphate (CaPs) materials [3,4,5]. One of the most studied CaPs is pure hydroxyapatite [HAp, (Ca_10_ (PO_4_)_6_ (OH)_2_], especially for the development of osteoconductive ceramic materials with application in orthopedics, dentistry, and general surgery [3,4,5,6,7,8]. Despite excellent biocompatibility and osteoconductive properties, pure HAp has limited applicability because of its inadequate mechanical properties (e.g., reduced strength and fragility) [9,10]. Another deficiency of HAp was reported to be the design limitations and the high degree of crystallinity which prevents resorption during bone remodeling stage [11,12]. Therefore, before designing a biocompatible device for the purpose of substituting human bone, the mechanical properties such as hardness, Young’s modulus, and fracture toughness need to be considered [9,10,11,12]. Synthetic HAp is structurally very similar with the human bone tissue and has remarkable osseointegration ability, which facilitates the formation of a strong bond with the bone. During the years, many studies have been focused on the improvement of mechanical properties, osteointegration, and implantation efficacy of apatitic bone substitutes [3,13,14].

Recently, researchers have turned their attention towards the effects induced by the single ionic substitutions on the HAp lattice [15,16,17,18,19]. Due to the apatitic structure, HAp can accommodate several anion and cation substitutions (e.g., F^−^, Cl^−^, Na^+^, K^+^, Mg^2+^, Sr^2+^, Ba^2+^, Al^3+^, Mn^2+^, Cu^2+^, Zn^2+^, Ag^+^, Ce^3+^, Eu^3+^, Sm^3+^), and furthermore can incorporate various types of complex ions (SiO_4_^4−^, HPO_4_^2−^, CO_3_^2−^) within its lattice [15,16,17,18,19,20]. The incorporation of these ions in the HAp lattice will allow for the modification of the crystallinity, lattice parameters, dissolution kinetics, and also other physical properties of the bioceramic [21]. Furthermore, the use of metal ions as dopants can determine an augmentation of HAp interactions with the surrounding cells, and can lead to the increase of HAp mechanical properties [22,23]. Magnesium (Mg) is a fundamental element and is the ninth most abundant element in the Earth’s crust [24]. Mg has an important role in the calcification process, helps reducing bone fragility, and has an indirect influence on mineral metabolism. It has also been reported to have a significant contribution in preventing possible risk factors of osteoporosis in humans [17,25,26,27]. Mg ions play an important role in the bone mineralization process influencing osteoblast and osteoclast activities [28,29]. The use of Mg-doped HAp has a considerable importance for the development of artificial bone substitutes. This is owned to the fact that Mg^2+^ combined with biological calcium phosphates exhibit an important role during the spontaneous formation of in vivo bone bonding, thereby being closely associated with the mineralization of calcified tissues [9,30,31]. Moreover, Mg^2+^ ions influence the mineral metabolism [31], and a shortage of Mg^2+^ ions has considerable adverse effects on all stages of the skeletal metabolism development. The lack of Mg in the human body could lead to severe affections of the organism, such as discontinuation of bone growth, reduce activity of osteoblasts and osteoclasts, osteopenia, and bone fragility [31,32]. It has been reported that in most of the calcified tissues, the amount of Mg associated with an apatitic phase is always higher at the beginning of the calcification process and has a significant decrease with the calcification increase. Moreover, studies have evidenced that Mg is a key factor in the qualitative development of the bone matrix that leads to bone fragility [28,32]. Even though these properties render magnesium and its composites suitable candidates for biomedical applications, especially for use as orthopedic materials, a low corrosion resistance in the physiological environment of these materials has been reported in the literature, which could lead to in vivo toxicity [33,34].

Previous studies regarding magnesium-doped hydroxyapatite have revealed that Mg ions inhibited the HAp crystallization in solution, and also that Mg in high concentrations could destabilize the structure of HAp [35,36]. Furthermore, studies have emphasized that Mg addition exhibited an inhibitory effect on HAp nucleation, its crystal growth, and also that Mg in high concentration causes a significant reduction in crystallinity coupled with an increase of its dissolution [37,38,39,40,41]. The complex investigation study of physico-chemical properties of hydroxyapatite doped with magnesium in solution [2,3,9,14,17] are rather limited.

In this context, this work aims to provide insights on the stability of suspensions of MgHAp nanoparticles synthesized by an adapted chemical co-precipitation method. Such information could contribute to better understanding of the surfaces of implantable materials and could help the identification of alternative solutions which can facilitate an improved interaction between CaPs and bone mineral implants. Non-destructive ultrasound-based technique was used for the first time to assess the stability of the MgHAp suspensions. Supplementary, ζ-potential, particles dispersion, and their hydrodynamic diameter were evaluated (D_H_). Moreover, the morphology, short-range and long-range order structure, and the inhibitory effect of 10MgHAp (x_Mg_ = 0.1) suspension against different microbial strains were also investigated. The results were interpolated and discussed.

## 2. Materials and Methods

### 2.1. Sample Preparation

#### 2.1.1. Materials

The synthesis of Ca_10−x_Mg_x_(PO_4_) _6_(OH)_2_, was conducted using calcium nitrate tetrahydrate, Ca(NO_3_)_2_∙4H_2_O (Sigma Aldrich, St. Louis, MO, USA, ≥99.0%), ammonium hydrogen phosphate, (NH_4_)_2_HPO_4_ (Sigma Aldrich, St. Louis, MO, USA, ≥99.0%) and magnesium nitrate hexahydrate, Mg(NO_3_)_2_·6H_2_O (Alpha Aesar, Germany, 99.97% purity), ammonium hydroxide, NH_4_OH [Sigma Aldrich, St. Louis, MO, USA, 25% NH_3_ in H_2_O (T)], ethanol absolute, C_2_H_6_O (Sigma-Aldrich, Sigma Aldrich, St. Louis, MO, USA ≥99.8% purity) and double distilled water.

#### 2.1.2. Magnesium-Doped Hydroxyapatite (MgHAp) Solution

An adapted chemical co-precipitation method was used to obtain the magnesium-doped hydroxyapatite sample [19]. The synthesis of Ca_10-x_ Mg_x_ (PO_4_) _6_(OH)_2_ was performed at room-temperature and the amount of magnesium was x_Mg_ = 0.1. (Ca + Mg)/P molar ratio was set to the stoichiometric value of 1.67 [15,18]. The solution obtained by dissolving Ca (NO_3_)_2_·4H_2_O and Mg(NO_3_)_2_·6H_2_O in a beaker was added dropwise into a solution of (NH_4_)_2_·HPO_4_. By adding NH_4_OH, the pH of the solution was kept constant at 11 throughout the synthesis process. The resulting mixture was stirred for 12 h at 80 °C. After stirring, the solution was centrifuged and re-dispersed in deionized water. This operation was repeated four times. The final solution specimen shall be further referred as 10MgHAp. For the X-ray diffraction (XRD), scanning electron microscopy (SEM) and energy dispersive X-ray spectroscopy (EDS) studies the solutions were dried in vacuum atmosphere and the obtained powders were analyzed.

### 2.2. Characterization Methods

ζ-potential and dynamic light scattering (DLS) analysis were effectuated at 25 ± 1 °C using a SZ-100 Nanoparticle Analyzer from Horiba-Jobin Yvon (Horiba, Ltd., Kyoto, Japan). The ζ-potential was measured after the sample was diluted 10 times. DLS measurements were also performed on the diluted sample. The 10MgHAp solution was placed in the disposable cuvette. The sample was illuminated with a laser source that allowed the estimation of the particle diffusion velocity to determine the hydrodynamic diameter (D_H_), which represents the particle diameter and also the molecules and/or ions which could be attached to the surface. The measurements were made in triplicate and the final value was the result of the three measurements.

**Figure 1:**
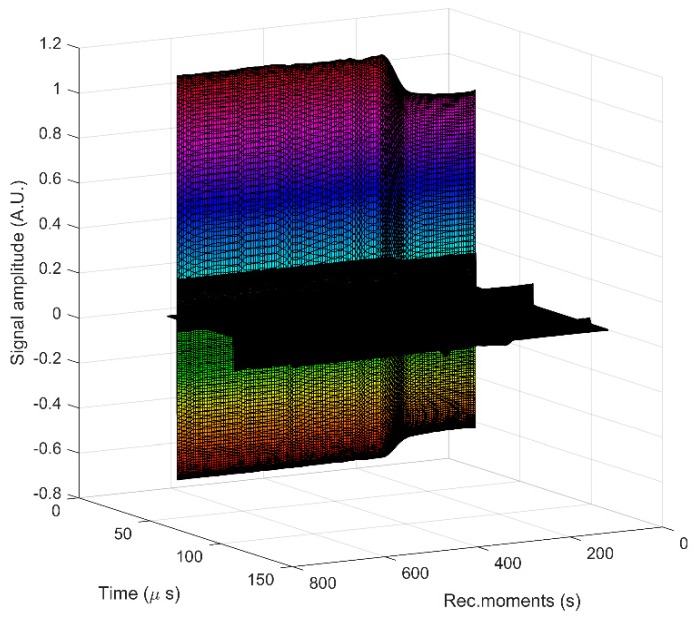
Recorded signals at 5 s recording interval. The increasing amplitudes of the first echo is visible in colors, whereas the second and third echo are significantly weaker (in black).

In order to perform the ultrasound measurements, the 10MgHAp suspension was stirred for 30 min at room temperature for a good homogeneity. The stirring process was conducted in a beaker containing 100 mL of suspension using a magnetic stirrer. The ultrasonic pulses were sent through the 10MgHAp suspension immediately after the stirring stopped. The experimental setup is described in detail in [42]. Essentially, two coaxial ultrasonic transducers held at a fixed distance are immersed in the suspension. The ultrasonic signals from the oscilloscope were recorded as digital files at a very precise interval of 5.00 s using a proprietary electronic device (Figure 1). This superposition of recorded signals indicates an increase of amplitudes with increasing recording moments. This evolution is relatively slow during the sedimentation process and is analyzed in the following. The only fast variation of amplitudes takes place in the interval 150–210 s, when the separation surface between the suspension and the solvent passes in front of the transducers. The second and third echoes are less visible as black peaks on this plot, being of much weaker amplitudes. These echoes are better viewed in the relative amplitudes evolution vs. the recording moments. In this manner, the evolution in time of the signals provides information about the stability of the suspension and attenuation vs. time. The signals processing is based on a comparison with the properties of double distilled water, taken as reference fluid, in the same experimental conditions.

The morphology of the 10MgHAp sample was investigated by scanning electron microscopy (SEM) with a HITACHI S4500 equipment.

The chemical composition of the bioceramic was assessed by energy dispersive X-ray spectroscopy (EDS) at 20 kV using an EDS system coupled to the SEM apparatus.

The investigation of the short-range order, structural bonding vibrations, and incidence of functional groups has been performed by Fourier-transform infrared (FTIR) spectroscopy in attenuated total reflectance (ATR) mode, using a Perkin Elmer Spectrum BX II spectrometer equipped with a Pike-MIRacle ATR head with diamond-ZnSe crystal. The FTIR-ATR spectra were collected in the wave numbers range 500-4000 cm^−1^ and represent the average of a total of 32 scans performed at a resolution of 4 cm^−1^.

The crystalline quality of the bioceramic was evaluated by X-ray diffraction (XRD), in Bragg–Brentano geometry with a Bruker D8 Advance diffractometer, using Cu K_α_ (λ = 1.5418 Å) radiation and a high efficiency LynxEye™ linear detector. The powdered materials were analyzed in the 2 θ range 15°–75° with a step size of 0.02° and an acquisition time per step of 5 s. The average crystallite size and lattice parameters were extracted by Rietveld whole powder pattern fitting [43] performed with MAUD v2.31 software. The crystallite shapes were modelled by applying the Popa approach [44], included in the MAUD software, which provides also their graphical representations.

### 2.3. Antimicrobial Assays

The antimicrobial activity of the 10MgHAp and HAp samples were investigated using both gram-positive (*Staphylococcus aureus* ATCC 25923, *Enterococcus faecalis* ATCC 29212), gram-negative (*Escherichia coli* ATCC 25922, *Pseudomonas aeruginosa* ATCC 27853), and fungal (*Candida albicans* ATCC 90029) reference strains. The quantitative antimicrobial assays were performed through serial microdilution method (96 well plates) in liquid medium. A volume of 10 µL of microbial suspension with a density of 10^6^ CFU/mL (CFU-colony forming units) was seeded in the wells. For each test, a microbial control culture (the microbial suspension grown freely in the culture medium) was also used. The microbial cells were grown in 96-well plates with nutrient broth in the presence of the tested samples and then incubated at 37 °C for 24, 48, and 72 h. Afterwards, the plates were washed and treated with methanol to fix the adherent cells. The biofilm formed on the walls of the wells was evidenced by staining with purple crystal solution and resuspension in acetic acid. The quantification of the influence of the 10MgHAp and HAp samples on biofilm formation was accomplished by reading the absorbance at 492 nm. The experiments were done in triplicate and the results were presented as mean ± standard deviation (SD).

## 3. Results and Discussions

**Figure 2:**
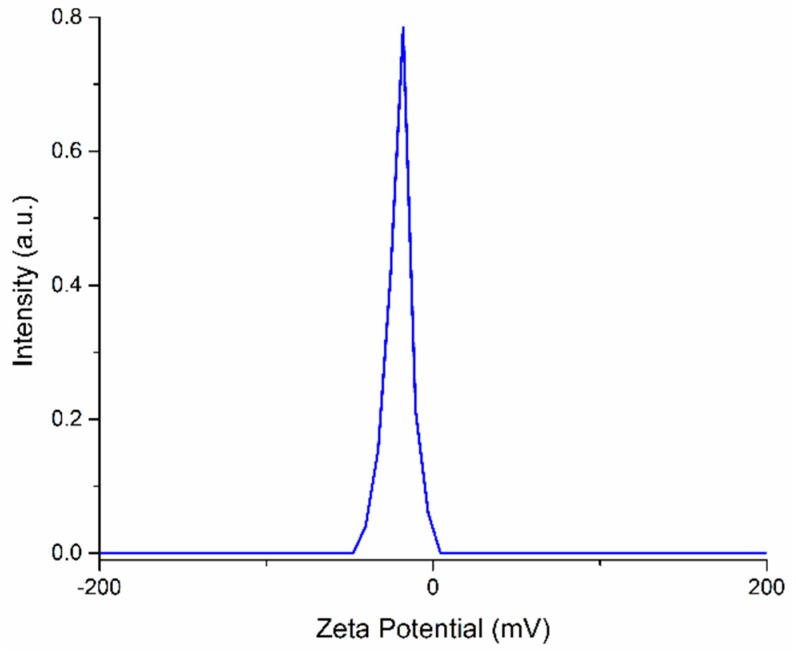
Zeta potential distribution for magnesium-doped hydroxyapatite (Ca_10−x_Mg_x_ (PO_4_)_6_ (OH)_2_, x_Mg_ = 0.1) (10MgHAp) nanoparticles suspension.

**Figure 3:**
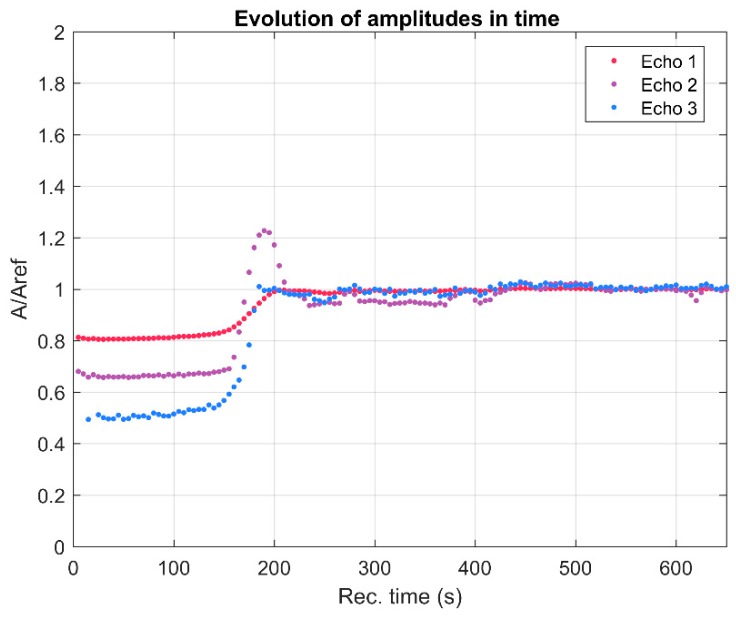
Relative amplitudes evolution vs. the recording moments.

**Figure 4:**
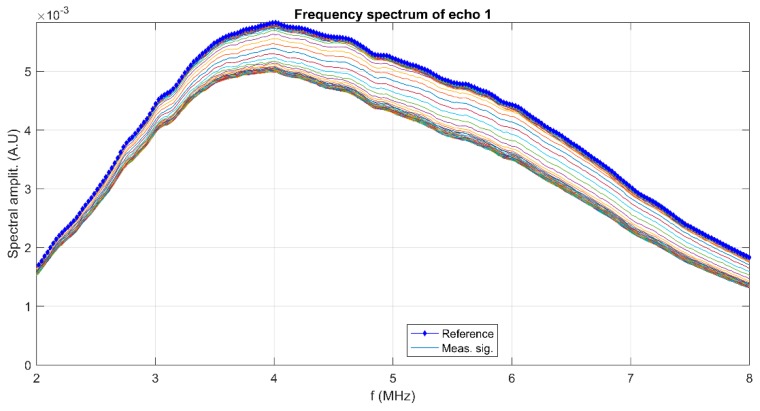
Frequency spectrum of the first transmitted echo for the reference fluid (◊) and all recorded signals (-).

**Figure 5:**
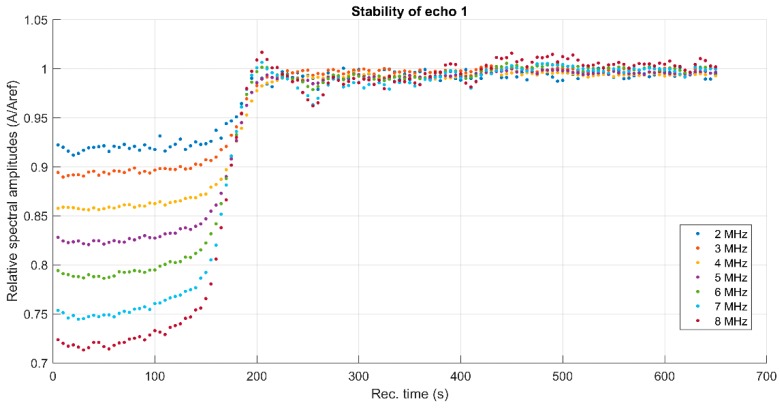
Spectral amplitudes relative variation vs. time, for the first echo.

**Figure 6:**
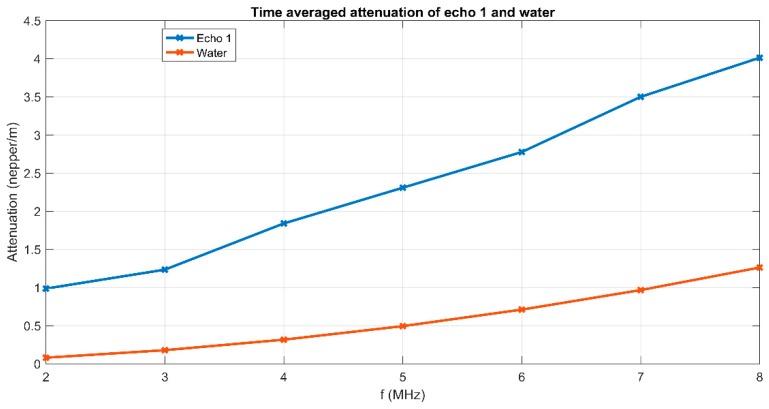
Attenuation vs. frequency for the first transmitted echo of the suspension (blue) and the reference fluid (red).

**Figure 7:**
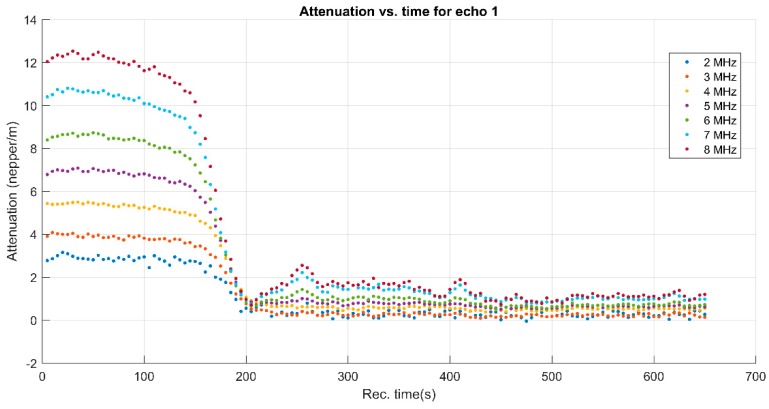
Attenuation vs. time for the spectral components of Echo 1.

**Figure 8:**
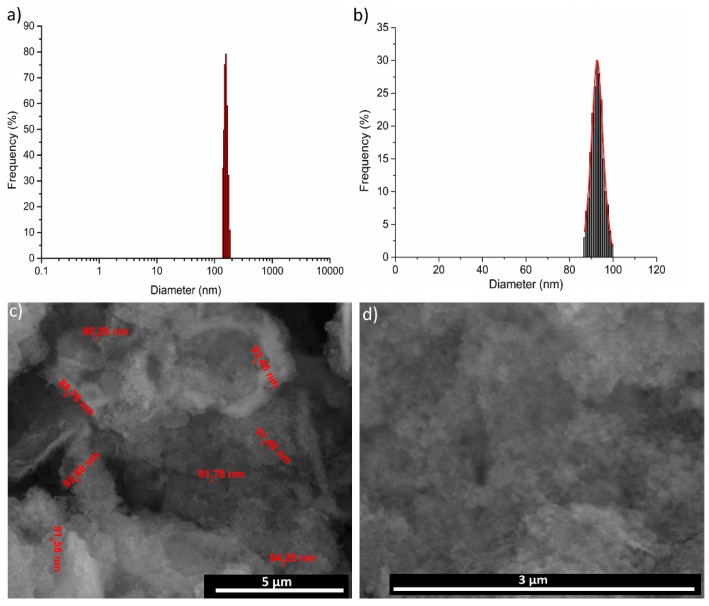
(**a**) The mean particle size and particle size distribution of the 10MgHAp sample, as determined by dynamic light scattering measurements. (**b**) The histogram of the particle size distribution. (**c**) scanning electron microscopy image with particle size. (**d**) Representative scanning electron microscopy image at a high magnification of the 10MgHAp nanostructured powder.

To cover the surfaces of implantable materials (e.g., orthopedic implants), the stability of the solutions to be used plays a very important role. Suspensions of 10MgHAp nanoparticles have been evaluated in terms of stability by ζ-potential (Figure 1), non-destructive ultrasound-based technique (Figure 2, Figure 3, Figure 4, Figure 5, Figure 6 and Figure 7), and dynamic light scattering (Figure 8a). In spite of its simplicity, the chemical co-precipitation method involves several parameters that can influence the quality of the suspensions.

One estimation of stability of MgHAp nanoparticles suspension was realized by ζ-potential measurements. The ζ-potential distribution obtained for 10MgHAp nanoparticles suspension is presented in Figure 2. The measured ζ-potential value for MgHAp nanoparticles in water was −17.77 ± 3.4 mV. The “low” value of the ζ-potential indicates that the suspension is unstable. According to previous studies [45,46,47], the colloids with low ζ-potential tend to flocculate, and indicate an incipient instability.

However, a “high” value of the ζ-potential does not provide a guarantee of the stability of the solutions. This is due to the fact that the ζ-potential measurements are made on unstable systems (i.e., at rest) [48]. Another drawback of this method is that the analysis is performed on diluted solutions. Thereby, it was decided to analyze the stability of these solutions using ultrasound measurements. The advantage of studying the stability of solutions by ultrasound measurements is given by the fact that the measurements were made on the initial solution (concentrated).

The stability assessment of 10MgHAp suspensions by ultrasound measurements was performed taking into account the most important parameters involved in determining the degree of stability, such as the ultrasonic velocity in the sample, the maximum amplitudes of the signals transmitted at the recording moments, and the frequency spectrum of the first transmitted echo. The most eloquent parameter with respect to the assessment of the stability of the nanoparticles suspension of 10MgHAp is the spectral amplitudes variation during the experiment. For a conclusive evaluation, the results obtained on 10MgHAp suspensions were compared with those obtained for water (which is a stable fluid) under the same experimental conditions. Time delays between the first three recorded echoes and those of the reference allow an accurate determination of the velocity of ultrasounds in the sample, for each recorded signal. The result is c = 1503.91 m/s, compared to the velocity in the reference fluid c_0_ = 1503.68 m/s at 27 °C. Because this velocity has a very small variation during the particle sedimentation it cannot be used to characterize the sample evolution in time. The maximums in transmitted signals amplitudes vs. recording moments (Figure 3) represent a significant variation. It has been observed that there is a slow variation for t < 150 s, during the sedimentation period, variation that will be used for further processing. The slow variation for t < 150 s was followed by a rapid variation of these amplitudes as the apparent surface of sedimentation passes in front of the coaxial transducers. After this passage, follows another a sedimentation period of scattered nanoparticles, in which the echoes amplitudes tend to those in the reference fluid. Since the second and third echoes are affected by ultrasound scattering, especially in the rapid transition period (150–210 s), producing even higher amplitudes than in the reference fluid (A_ref_), we decided to use only the first echo in the following analysis (Echo 1 in Figure 3).

The first period (t < 150 s) is relevant for the suspension stability. The amplitude of the first echo, which is measured with highest accuracy, has a slowly increasing value during this period. The slope of this amplitude vs. time is related to the stability parameter, which in this case is $s=\frac{1}{A_{m}}\left|\frac{dA}{dt}\right|$ = 0.087 (1/s), with *A_m_* the averaged amplitude of the signals during this sedimentation period. This value of the s parameter is typical for a relatively weak stability. Another characteristic of the suspensions is represented by the frequency spectrums of the first transmitted signals, using the Fourier-transform of the first echo at all recording moments (Figure 4). The lowest curves correspond to the first period of the monitored sedimentation (t < 150 s) and the upper ones correspond to the asymptotic sedimentation of the suspension fluid, tending to evolution of the reference fluid. A curve is plotted for the first echo at each recorded moment, from the first (lowest curve) to the last recorded moment (highest curve). The peak at 4 MHz is a characteristic of the transducers. The larger differences relative to the reference fluid (marked by ◊), especially between 4 and 7 MHz, indicate a higher absorption of acoustic energy by the dispersions at different recording moments, due to the concentration and size of the nanoparticles. Combined information of the previous two figures is the spectral amplitudes variation during the experiment. In Figure 5, is shown to be the highest amplitude ratio of 0.92 at 2 MHz, which decreases to less than 0.75 at 8 MHz, indicating a higher absorption of acoustic energy with increasing frequency. After the rapid transition period, these amplitudes tend to 1 in an oscillatory manner, due to various sedimentation velocities as a function of particle dimensions.

A more explicit information on the attenuation dependency of frequency (in blue) is given in Figure 6, compared to the same dependency on frequency for the reference fluid (in red). The attenuation is considerably higher (1–4 nepper/m) for the sample compared to 0.3–1.3 nepper/m for the reference fluid. Certainly, the attenuation for each spectral component depends on the moment during the experiment, as shown on Figure 6. During the initial stage, an attenuation of 12 nepper/m for the 8 MHz component in the recorded Echo 1 is a characteristic of this sample, as shown in Figure 5. The lowest attenuation of 3 nepper/m is obtained for the 2 MHz spectral component. During the asymptotic sedimentation period (after 200 s in this case), the attenuation coefficients tend towards stable values, which correspond to those in the reference fluid. As a partial conclusion, the first transmitted echo, recorded during the recording interval 100–150 s provides sufficient information for a complete characterization from the ultrasonic point of view of this sample: stability, overall and spectral attenuation.

The hydrodynamic diameter (D_H_) of the 10MgHAp nanoparticles were determined by DLS measurement of the particle size. The relationship between particle size and Brownian motion is described by the Stokes–Einstein Equation:

$$D_{H}=\frac{\mathrm{kT}}{3\pi \eta C},$$

where, *D_H_* = hydrodynamic diameter, *k* = Boltzmann constant, *T* = absolute temperature, *η* = dynamic viscosity of the solvent, and *C* = diffusion constant.

The average particle size and particle size distribution of the 10MgHAp sample analyzed by DLS method are presented in Figure 8a. The hydrodynamic diameter obtained was 158.3 ± 18 nm. A representative SEM image of the 10MgHAp material is presented in Figure 8c. The 10MgHAp particles have an approximately round shape and tend to aggregate into larger clusters. In order to determine the size distribution of 10MgHAp nanoparticles, the diameters of approximately 500 nanoparticles was measured (Figure 8c). The histogram reflecting the resulting values was fitted with a Gaussian function (Figure 8b). The average particle size calculated from SEM (D_SEM_) image was of 93.3 ± 8 nm (Figure 8b). Moreover, a SEM image at a higher magnification was presented in Figure 8d.

**Figure 9:**
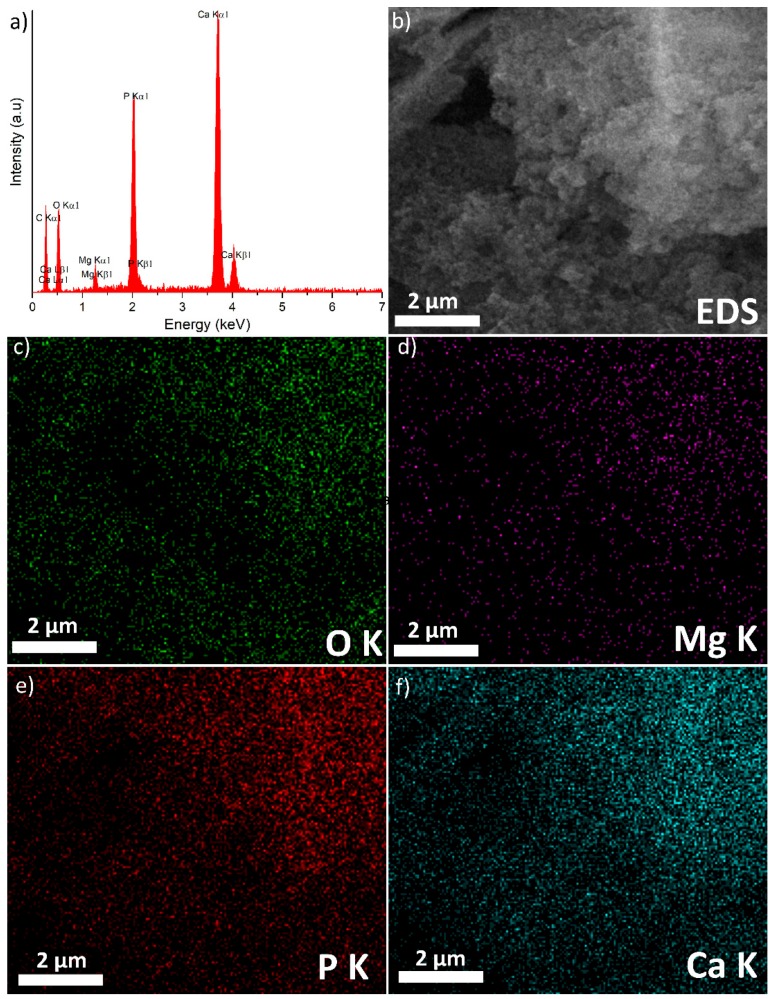
Energy-dispersive X-ray spectroscopy spectra (**a**), scanning electron microscopy image used for the energy-dispersive X-ray spectroscopy analysis (**b**), elemental mapping analysis (**c**–**f**) of the 10MgHAp.

The chemical composition and uniform distributions of the 10MgHAp sample were evaluated by EDS studies (Figure 9) on a randomly-selected area of the specimen (pictured in the SEM images presented in Figure 9b). Only the peaks of the typical chemical elements characteristic to the 10MgHAp sample (i.e., Ca, P, O, Mg) were observed in the EDS spectrum (Figure 9a), testifying for the material good purity. The presence of carbon in the EDS spectra could be due to the experimental procedure of the analyses and to the common impurities from the HAp synthesis. Furthermore, an elemental mapping analysis (Figure 9c–f) was performed on this selected microscopic field (Figure 9b). The elemental mapping indicated the uniform and homogenous distribution of O, Mg, P, and Ca in the 10MgHAp specimen (the apparent different color hues are due to nonhomogeneous height distribution of the sample (composed of randomly-agglomerated powder particles)).

Furthermore, the structural characteristics of 10MgHAp were evaluated by FTIR-ATR and XRD analyses.

**Figure 10:**
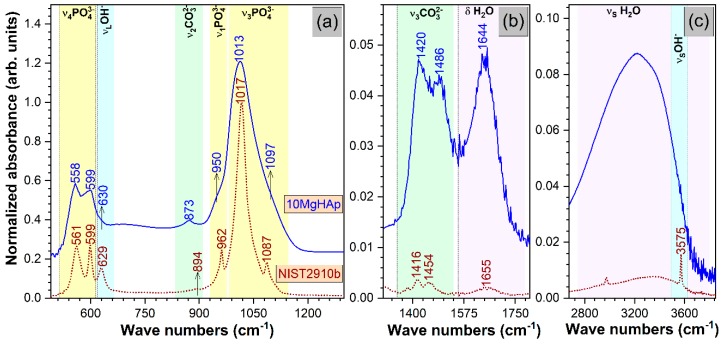
The comparative Fourier-transform infrared (FTIR) spectroscopy in attenuated total reflectance (ATR) mode spectra of the 10MgHAp and National Institute of Standards and Technology SRM2910-b samples in three relevant wave number regions: (**a**) 500–1300 cm^−1^; (**b**) 1300–1800 cm^−1^; and (**c**) 2700–3800 cm^−1^.

The comparative FTIR-ATR spectra of the 10MgHAp and NIST (National Institute of Standards and Technology) SRM (Standard Reference Material) 2910b samples are displayed in Figure 10. The 10MgHAp sample elicited broader IR bands with less conspicuous peaks when compared to the well-crystallized pure hydroxyapatite NIST SRM2910b specimen or to a pure HAp powder synthesized under similar conditions [49,50], which suggests that important structural distortions are inflicted by the large concentration of cationic substitution introduced into the HAp lattice. However, all the vibration bands characteristic to a HAp material were evidenced [51], although presenting slight shifts as a consequence of the aforementioned network/bonding modifications: Triply degenerated out-of-plane bending ν_4_ (~500–610 cm^−1^), symmetric stretching ν_1_ (~950–962 cm^−1^), triply degenerated asymmetric stretching ν_3_ (~980–1100 cm^−1^) of orthophosphate units, and liberation (~620–640 cm^−1^) and stretching (~3575 cm^−1^) of structural hydroxyl groups (Figure 10a,c). With respect to the NIST SRM2910b, the 10MgHAp bioceramic contained a higher amount of adsorbed water (to be expected for a compound synthesized by a wet-chemistry technology), as evidenced by the more intense bands associated to the characteristic bending (~1630–1670 cm^−1^) and stretching (~2700–3700 cm^−1^) vibrations of water molecules (Figure 10b,c). The shallow band peaking at ~2900 cm^−1^ in the case of NIST-SRM 2910b appertains to various C–H stretching vibrations, the consequence of an accidental minor sample contamination during storing or handling [52]. Furthermore, it should be emphasized that the 10MgHAp is also carbonated (Figure 10a,b). The positions of the bending ν_2_ (~873 cm^−1^) and asymmetric stretching ν_3_ (~1416 and 1454 cm^−1^) are advocating for a B-type carbonatation, with the carbonate groups substituting the orthophosphate groups in the HAp lattice [51]. The B-type carbonatation is found to be prevalent in bone mineral [53], and it is recognized to possess superior biological performance with respect to pure HAp [54,55,56].

**Figure 11:**
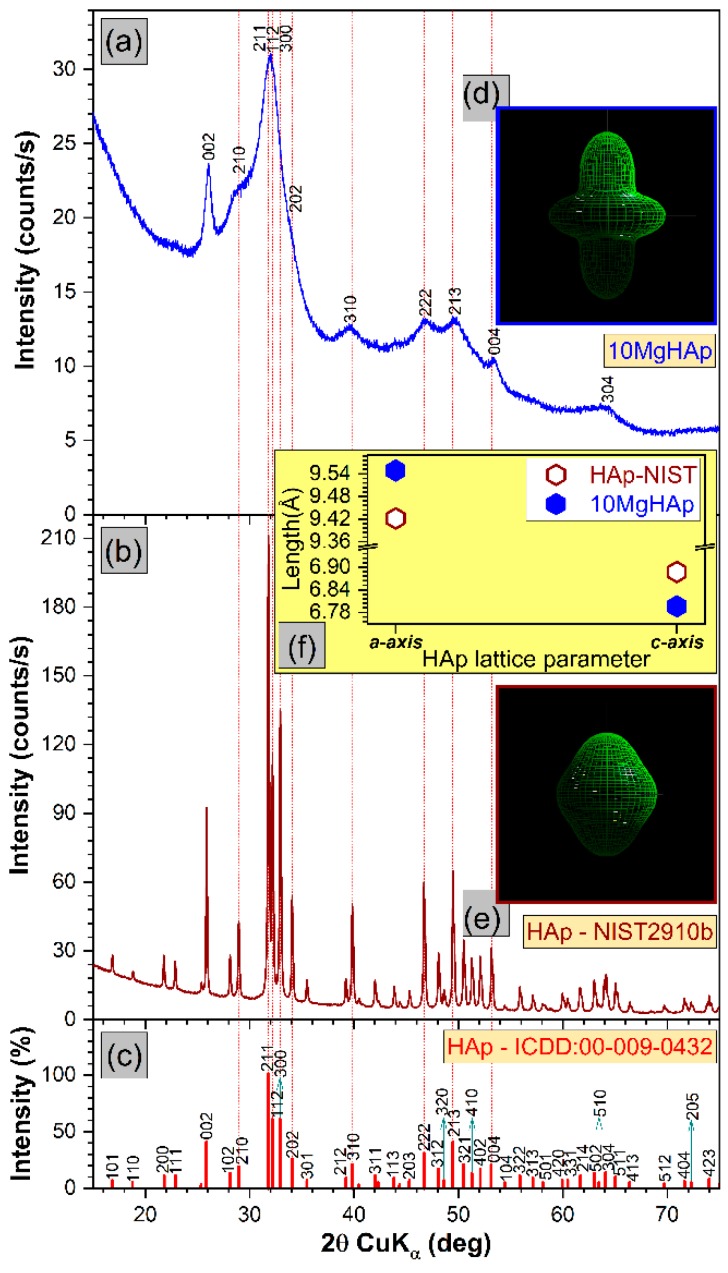
The comparative XRD patterns of the (**a**) 10MgHAp and (**b**) NIST SRM2910-b samples with respect to the (**c**) ICDD-PDF4: 00-009-0432 reference file of hexagonal hydroxyapatite. Crystallite shape as determined by MAUD modelling for (**d**) 10MgHAp and (**e**) NIST SRM2910-b. (**f**) The *a*-axis and *c*-axis lattice parameters of the 10MgHAp sample with respect to the ones determined for NIST SRM2910-b.

The comparative XRD patterns of 10MgHAp and NIST SRM2910b pure HAp reference samples are presented in Figure 11. All the diffraction maxima recorded in the case of the 10MgHAp sample are associated to a hexagonal hydroxyapatite phase (ICDD-PDF4: 00-009-0432), namely to the 002, 210, 211, 112, 300, 310, 222, 213, 004, and 304 crystal plane reflections. There was no evidence (at the sensitivity limit of the machine) of residual phases or segregated compounds. The broad and convoluted allure of the 10MgHAp peaks (Figure 11a) with respect to the NIST SRM2910b reference sample (Figure 11b) indicates the nanosized nature of the magnesium-doped sample, as a consequence of the large Mg^2+^ cation concentration incorporated into the HAp lattice. This is in good agreement with the FTIR-ATR spectroscopy observations.

In order to determine the microstructural characteristics of HAp by Rietveld whole pattern fitting, an anisotropic crystallite-sized model must be considered, as an evidence that the crystallite shapes are not isotropic. We used the Popa anisotropic model [44] implemented in the MAUD software. The shape of “the composite crystallite” that resulted from modeling is represented in Figure 11d,e. For isotropic crystallites the shape of the “composite crystallite” should be spherical. The results indicated that the NIST SRM2910b sample consisted of quasi-spherical crystallites (Figure 11e) with a mean diameter of ~90 nm, with *a*-axis and *c*-axis lattice parameters (i.e., 9.421 Å and 6.887 Å) situated in the close vicinity of the ones listed by ICDD-PDF4: 00-009-0432 reference file (i.e., 9.418 Å and 6.884 Å). Nevertheless, the substantial Mg substitution is inducing the increase of the *a*-lattice constant (i.e., 9.54 ± 0.06 Å), accompanied by the decrease of the *c*-lattice constant (i.e., 6.80 ± 0.03 Å) (Figure 11f). Similar observations on the evolution of MgHAp lattice constants were reported by Bigi et al. [39]. The fitting of the 10MgHAp pattern suggested that this material is indeed composed of nanosized, disordered crystallites (mean length of ~3.6 nm), most probably both elongated and flattened along the *c*-axis (Figure 11d). One should note that a dramatic reduction of the average crystallite size is recorded for the Mg-doped HAp with respect to the pure HAp powder (i.e., having a mean crystallite size of ~23 nm) synthesized under similar conditions [50]. Thereby, the ~93 nm large particles, visualized by SEM in the case of the 10MgHAp samples, are composed, on average, of around 25 crystallites. However, the quantitative Rietveld evaluation in the case of the 10MgHAp should be considered as indicative only, since the fitting was not entirely satisfactory in spite of all dedicated efforts.

The HAp doping with 10 at.% Mg significantly influenced the structure of the ceramic, as shown by XRD and FTIR-ATR results, determining the formation of an ultra-nano-crystalline material. The particle size obtained from DLS (D_H_) was bigger than the size obtained by SEM (D_SEM_). While SEM, FTIR-ATR, and XRD are solid state analysis methods, DLS is a dispersions investigation technique and is based on the measurement of intensity fluctuations caused by the interference of laser light (that is scattered) through diffusing particles. The difference between the SEM and DLS results could be due the fact that SEM cannot measure any particle coating agent, while DLS provides information about the hydrodynamic diameter (i.e., the particle diameter) plus the molecules or ions that are attached to its surface [57]. Thus, the molecules or ions that are attached to the surface of the particles make the particle seem larger, which is why the hydrodynamic diameter is always greater than the particle size estimated by SEM [58]. The difference between D_H_ and D_SEM_ of 10MgHAp could be explained by both the agglomeration of the particles in the flocculation process and the particle surface water layer. In the case of 10MgHAp suspensions, the surface of the particles created an affinity for water which led to the creation of a structured water layer on the surface of the particles. On the other hand, particle agglomeration could be an explanation for dispersal instability, as highlighted by ζ-potential results, and further confirmed by ultrasound measurements. In agreement with previous studies [59], the average size of nanoparticles plays an important role in material performance such as solubility, dissolution, stability, and cellular absorption.

**Figure 12:**
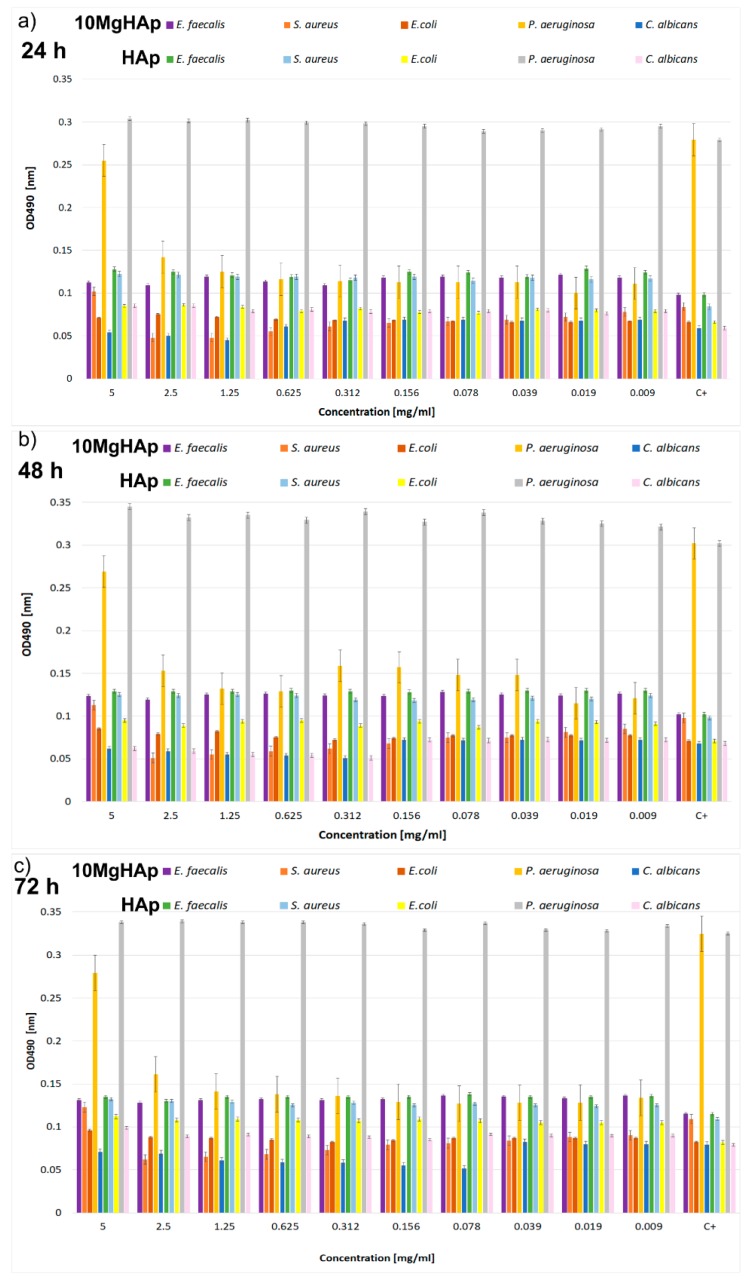
The dynamics of the antimicrobial activity of 10MgHAp and HAp against *Enterococcus faecalis* ATCC 29212, *Staphylococcus aureus* ATCC 25923, *Escherichia coli* ATCC 25922, *Pseudomonas aeruginosa* ATCC 27853, and *Candida albicans* ATCC 90029 microbial cells after (**a**) 24 h, (**b**) 48 h, and (**c**) 72 h of incubation.

Recently, the antimicrobial effect of metallic or inorganic nanoparticles such as copper, iron, silver, magnesium, and zinc against numerous clinical or reference microbial strains have been evaluated in several in vitro studies [42,60,61,62,63,64,65,66,67]. Our study highlights, for the first time, the influence of magnesium-doped hydroxyapatite solutions on the development of microbial biofilms. The influence of the 10MgHAp suspensions on the biofilm formation of gram-positive (*Staphylococcus aureus* ATCC 25923, *Enterococcus faecalis* ATCC 29212) reference strains, gram-negative (*Escherichia coli* ATCC 25922, *Pseudomonas aeruginosa* ATCC 27853) reference strains, and a fungal (*Candida albicans* ATCC 90029) reference strain was assessed at three different time intervals (24, 48, and 72 h). Moreover, the antimicrobial effects of HAp suspensions were also investigated using the same microbial strains. The results of the antimicrobial assays are depicted in Figure 12a–c. Studies regarding the influence of the tested samples against microbial biofilm formation have emphasized that 10MgHAp suspensions exhibited excellent antimicrobial properties even after 24 h in the case of gram-negative *P. aeruginosa* and gram-positive *S. aureus* bacterial strains (Figure 12a), comparative to both the control and HAp suspensions. The results of the antimicrobial assays highlighted that HAp suspensions had the tendency to facilitate the biofilm formation of all the investigated microbial cells for all the tested concentrations and at all tested time intervals (Figure 12a–c). The values of the absorbance at 490 nm obtained in the case of the microbial cells incubated with HAp suspensions were comparable or higher to the ones obtained for the control culture (the culture grown in the absence of the samples). These results indicate that the antimicrobial effect of the 10MgHAp suspensions are due to the magnesium presence in the lattice of the hydroxyapatite. The 10MgHAp solution also inhibited the biofilm formation of *P. aeruginosa* for all tested concentrations ranging from 5 to 0.009 mg/mL, while in the case of the *S. aureus* bacterial strain, the inhibition was recorded starting with a concentration of 2.5 mg/mL. On the other hand, the HAp suspensions promoted the biofilm formation of *P. aeruginosa* and *S. aureus* bacterial strains for all tested concentrations, even after 24 h of incubation, relative to control, having noticeable higher values of OD_490_ nm than the control. Moreover, a moderate inhibition of the biofilm formation was also noticed after 24 h in the case of the *C. albicans* fungal strain for concentrations ranging from 5 to 1.25 mg/mL of the 10MgHAp suspensions (Figure 12a). *E. faecalis* and *E. coli* biofilm development was not influenced by the presence of 10MgHAp samples at any of the tested concentrations. More than that, HAp suspensions had a stimulating effect on the *E. faecalis* and *E. coli* biofilm development. The quantification values obtained in the case of *E. faecalis* and *E. coli* biofilm development were comparable to the ones of the control, or had only hardly noticeable lower values in regard to the control, but were observable lower than the ones obtained in the case of HAp suspensions. The same comportment was observed at 48 and 72 h. Furthermore, the results of the antimicrobial assays evidenced that also, after 48 h of incubation, 10MgHAp suspensions exhibited an excellent inhibitory effect against biofilm development in the case of *P. aeruginosa*, *S. aureus*, and *C. albicans* microbial strains (Figure 12b), comparative to HAp suspensions and control. Moreover, it can be observed that after 48 h the range of concentration for which 10MgHAp suspensions inhibited the biofilm formation in the case of the *C. albicans* fungal strain was expanded up to 0.312 mg/mL (Figure 12b). After 72 h of incubation, the inhibition of biofilm development was also emphasized in the case of *P. aeruginosa*, *S. aureus*, and *C. albicans* microbial strains (Figure 12c). *P. aeruginosa* and *S. aureus* biofilm development was considerably inhibited for all tested concentrations, while the inhibitory effect range of *C. albicans* got as far as 0.078 mg/mL. The results have emphasized that the HAp suspensions had a stimulating effect on the biofilm development of all tested microbial strains, rendering the fact that the antimicrobial properties of the 10MgHAp samples could be entirely attributed to the magnesium presence in the hydroxyapatite lattice.

The antimicrobial properties of magnesium and magnesium composites have been attributed to various mechanisms, which are similar to the ones encountered in the case of other metallic ions. The literature revealed that nanoparticles have toxic effects on human organisms by triggering oxidative stress, inflammation, and even indirect DNA damage [68,69]. Moreover, metal oxide nanoparticles toxicity has been attributed to reactive oxygen species (ROS) production, which is responsible for inducing oxidative DNA damage, protein denaturation, and lipid peroxidation [70,71]. As for other metal oxide nanoparticles, the toxicity of magnesium oxide nanoparticles was also attributed to reactive oxygen species (ROS) production. Furthermore, it was confirmed that MgO nanoparticles release important amounts of magnesium ions but these ions did not present any significant toxicity [72,73]. Numerous studios have investigated toxic effects of MgONPs using fish as model organisms [72,73]. A study, [73], investigated the cytotoxicity of MgO NPs using tilapia and zebrafish, and demonstrated that magnesium oxide bulk particles presented a higher level of toxicity compared to magnesium oxide nanoparticles. Moreover, in this study it was revealed that no mortality was noticed in the case of zebrafish exposed to MgONPs, while a 100% mortality rate was obtained in the case of exposure to bulk particles. Another research paper, [69], also demonstrated that MgONPs (20 nm in size) exhibited a lethal effect on zebrafish embryos and that the survival of the zebrafish embryos decreased significantly with the increase of MgONPs, and concluded that the production of intracellular ROS was one of the main mechanisms of toxicity caused by MgONPs in zebrafish embryos. Nonetheless, all the studies presenting the toxicity of magnesium compounds are strictly related to the existence of magnesium oxide and toxicity is not attributed to magnesium alone. On the other hand, concerning the antimicrobial properties against different microbial strains, it has been reported that magnesium ions have the ability to infiltrate inside the bacterial cells of the microorganisms and create a disturbance of the membrane potential [62,65,66,67]. Furthermore, it was found that high concentrations of OH^−^ appeared on the surface of the nano-MgO particles and increased the O^2−^ concentration, which is known to aid a more pronounced destruction of the bacterial cell wall [67]. Moreover, the efficiency of different concentrations of nano-MgO aqueous solution, sodium hypochlorite (NaOCl), and chlorhexidine (CHX) gluconate against several resistant bacterial strains (*S. aureus*, *E. faecalis*) and a fungal strain (*C. albicans*), tested at different time intervals, was reported [65]. It was concluded that nano-MgO aqueous solutions exhibited a better activity against *E. faecalis* bacterial cells compared to NaOCl. Moreover, it was highlighted that the use of magnesium oxide nanoparticles lead to a prolonged antibacterial activity with respect to NaOCl [65]. The results regarding the antimicrobial activity of 10MgHAp suspensions obtained in this study emphasized that magnesium-doped hydroxyapatite solution is most effective in inhibiting biofilm formation for *P. aeruginosa*, *S. aureus,* and *C. albicans* microbial strains, even when used in low concentrations (0.009 mg/mL). Moreover, the results have evidenced that the inhibitory effect is dependent on the incubation time and also on the sample concentration in the case of 10MgHAp suspensions. According to [74,75], corrosion products released by modern biocompatible magnesium alloys do not significantly affect human metabolic pathways. Moreover, recent studies [76,77] have shown that implantation of a magnesium device causes minimal changes in blood composition without causing damage to the excretory organs, such as the liver or kidneys. In agreement with previous studies, the colloidal stability suspensions of hydroxyapatite doped with different cations were influenced by several parameters such as ζ-potential, spectral attenuation, amplitude of the first echo, or hydrodynamic diameters [15,18,78,79]. Therefore, the studies presented in this paper are of great interest for the development of cost-effective, stable, and reproducible antimicrobial agents for biomedical applications.

## 4. Conclusions

The magnesium-doped hydroxyapatite (Ca_10−x_ Mg_x_ (PO_4_)_6_ (OH)_2_, x_Mg_ = 0.1) suspensions were obtained by an adapted, simple, and low-cost chemical co-precipitation at room-temperature synthesis method. This study presented results obtained by both solid-state analysis techniques (SEM, FTIR-ATR, and XRD) and suspension-based analysis methods (ζ-potential, ultrasound measurements, and DLS). Based on the results obtained using complementary analysis techniques, the stability of the 10MgHAp suspensions, size of the nanoparticles, and structural status were determined. The analyses such as ζ-potential and DLS were performed on diluted solutions of 10MgHAp nanoparticles. The concentrated solutions of 10MgHAp were evaluated using ultrasound measurements. Both ζ-potential and ultrasound measurements showed a poor stability of 10MgHAp suspensions. FTIR-ATR and XRD measurements revealed that the obtained 10MgHAp nanoceramic had a high degree of purity (only the hydroxyapatite phase without other impurities). The uniform distribution of the constituent elements of MgHAp were demonstrated by elemental mapping EDS analysis. The 10MgHAp suspensions demonstrated antimicrobial efficacy against *P. aeruginosa*, *S. aureus*, and *C. albicans* microbial strains. The 10MgHAp suspensions could be used for the fabrication of biocompatible antimicrobial coatings of medical devices.

This study highlighted the possibility of obtaining suspensions of magnesium-doped hydroxyapatite and also the evaluation, for the first time, of the behavior of these suspensions, both from the physico-chemical and the biological point of views. The research concerning the stability of the 10MgHAp concentrated solutions were conducted for the first time by a non-destructive ultrasound based technique. In addition, the influence of the 10MgHAp particles suspension on the microbial biofilm development of different microbial strains was also investigated for the first time.

Further research will focus on increasing the stability of magnesium-doped hydroxyapatite suspensions to facilitate obtaining homogeneous coatings.
